# Case of Treatment-Resistant Aggression in the Context of a Medically Underserved Community

**DOI:** 10.7759/cureus.25817

**Published:** 2022-06-10

**Authors:** Sergio Trejo, Michelle I Malwane, Erica Y Kim, Eric B Nguyen, José R Cucalón-Calderón

**Affiliations:** 1 Department of Pediatrics, University of Nevada Reno School of Medicine, Reno, USA

**Keywords:** child and adolescent psychiatry, pediatrics, underserved, adhd, aggression

## Abstract

Aggression tends to decrease as a child matures and develops conflict resolution skills. However, aggression can persist if children are exposed to consistent negative stimuli, such as poor parenting and adverse childhood experiences (ACEs). Furthermore, aggression is commonplace in numerous psychiatric disorders, such as attention deficit hyperactivity disorder (ADHD) and oppositional defiant disorder. These negative stimuli and comorbid conditions could ultimately stunt a child’s development during pivotal moments, leading to worsening aggressive behaviors, such as criminal activity. Behavioral interventions are imperative for individuals with these comorbid conditions and experiences.

Our patient is an 11-year-old male with a pertinent past medical history of ADHD and disruptive mood dysregulation disorder (DMDD) on multiple psychotropic medications, who presented to the emergency department for the evaluation of homicidal ideation and suicidality. This also occurred with worsening aggressive behavior, demonstrated by his killing of family pets and subsequent threats to kill his family. The patient has had multiple emergency department visits for similar threats and has been admitted to numerous inpatient psychiatric facilities. Currently, the patient is being treated at an out-of-state inpatient psychiatric facility.

Our patient’s aggression most likely stems from his comorbid ADHD and DMDD, complicated by other factors, such as low socioeconomic status, limited access to mental health services, and living in a medically underserved community. Factors such as increasing primary care provider comfort in managing these conditions, especially in underserved communities where there are already shortages of mental health providers, could help address this issue. Furthermore, it is imperative to screen for other contributing factors, such as ACEs. This case also highlights the need for genetic testing as a part of the medical workup in psychiatric cases that display psychotropic medication resistance. However, genetic testing is something that is not readily available in our state and is not covered by Medicaid.

Early treatment of mental health conditions can prevent social difficulties later in life. With aggression, providing appropriate interventions is key to preventing an individual from engaging in harmful activities. It is important to screen for ACEs in order to address well-known aggravating factors. Underserved populations also face a myriad of challenges that prevent them from accessing healthcare services. There are numerous problems contributing to this disparity, ranging from lack of adequate mental health services to lack of access. Accordingly, it is imperative that primary care physicians and providers practicing in underserved areas receive the training necessary to recognize and treat mental health conditions. Furthermore, physicians should be able to focus on psychosocial stressors that contribute to these mental health conditions and provide the resources necessary to address these factors.

## Introduction

Among children and adolescents, expression of aggression ranges from roughhousing during games to name calling [1]. Frequently, boys tend to display more aggressive behaviors across the lifespan that range from fighting during school years to violent crimes as they transition into adulthood [1,2]. Tantrums, fighting, and other behaviors that violate social norms, such as lying, cheating, and breaking the law, are common manifestations of aggression [1]. Typically, however, aggression tends to decrease throughout development. While approximately 50% of social interactions between children over the age of 2 involve some form of conflict, this percentage decreases to 20% for children aged 12 to 18 [1]. The development of conflict resolution techniques without the use of aggression is key as a child becomes a member of society [1]. Despite this, maladaptive aggression patterns can develop if children are exposed to consistent negative stimuli such as ongoing toxic stress, inconsistent and poor parenting, peers who consistently break rules, and adverse childhood experiences (ACEs) [3]. Furthermore, aggression is a shared feature among several psychiatric conditions, especially those involving poor impulse control. These conditions include ADHD, oppositional defiant disorder, Tourette’s syndrome, intermittent explosive disorder, and disruptive mood dysregulation disorder (DMDD) [4,5]. Together, these negative stimuli and comorbid psychiatric conditions could stunt a child’s development during pivotal moments and lead to worsening aggressive behaviors. Emotional dysregulation and aggression can contribute to conduct disorder, which can ultimately become antisocial personality disorder [4]. Other adverse outcomes that can manifest include withdrawal from school, substance abuse, and difficulty in creating and maintaining social connections, all of which can decrease quality of life [5]. To prevent adverse psychosocial outcomes, it is imperative that individuals with these conditions are provided with early behavioral interventions with a focus on behavior-modifying strategies. These may include reward systems, participation in therapy sessions, learning age-appropriate social skills, problem-solving, and parent training [6]. These interventions can be combined with psychotropic medications to augment response.

## Case presentation

Our patient is an 11-year-old male with a past psychiatric history significant for ADHD and DMDD, managed by his psychiatrist, currently on 100 mg of amantadine, 0.2 mg of clonidine, 100 mg of quetiapine, 100 mg of trazodone, and 40 mg of lisdexamfetamine daily. The patient is currently on the maximum dosage for each medication. The patient's family history is pertinent for a brother with Asperger's syndrome and unspecified bipolar disorder, a paternal grandfather with ADHD, a paternal uncle with Asperger's and ADHD, a maternal grandmother with unspecified bipolar disorder, a maternal grandfather with schizophrenia, and a maternal uncle who committed suicide. Birth history is significant for a gestational age of 35 weeks alongside a twin sister who died in utero. The patient's mother denies any illicit drug use during her pregnancy; however, she did have a systemic lupus erythematosus flare, which required the use of steroids throughout the pregnancy. The patient has no history of any form of abuse. However, it is notable that the patient moved to Reno, Nevada, a few years prior due to a reportedly negative influence that the mother's extended family was having on the child. The patient's mother did not elaborate further on the nature of the influence. After this move, the patient started to experience worsening insomnia and aggression. In addition to seeing psychiatry, the patient is seeing a psychotherapist and also has an individualized education plan at school due to his behavior.

Our patient initially presented to the emergency department for the evaluation of homicidal ideation and increasingly aggressive behavior. The patient was brought to the emergency department by his mother, who had concerns about his behavior, which included poisoning and killing four kittens with lack of remorse. The following afternoon, the patient’s behavior escalated to the point that he threatened to kill both his mother and his older brother. The patient’s mother attempted to have him evaluated by mobile crisis hotline; however, they were unable to conduct the evaluation over the phone at the time. She then attempted to take him to an inpatient psychiatric facility, but the patient became more agitated and threatened to jump off a large drop-off. Due to imminent danger and need for immediate attention, the patient's mother decided to go to the emergency department instead. During the initial evaluation in the emergency department, the patient was running around the examination room, extremely agitated with pressured speech and flight of ideas. The patient continued to be aggressive during his stay in the hospital, as demonstrated by biting, punching, and yelling profanities to staff. This occurred multiple times throughout his stay, requiring sedation with his home medications, diphenhydramine, ketamine, and lorazepam. The patient's psychiatrist was consulted during this stay regarding concerns for using lisdexamfetamine at this time; however, they approved it since per the patient's parents, it improved similar symptoms in the past. Ultimately, it was deemed necessary to transfer patient to an inpatient psychiatric facility; however, due to the lack of beds available at the time of the patient's visit to the emergency department combined with a shortage of child and adolescent psychiatrists in the state, which makes it difficult to get an appointment, even with his own psychiatrist, it was determined that the patient would be sent to an out-of-state in-patient psychiatric facility.

Our patient has had multiple visits to the emergency department since the age of 9 for homicidal ideation involving his family as well as suicidal thoughts. These episodes were triggered by being told to do his homework or to go to bed and occurred while running away from home. In addition to the medications that he is currently taking, he has an extensive history of other psychotropic medication trials including, aripiprazole, risperidone, amphetamine-dextroamphetamine, methylphenidate, dexmethylphenidate, fluoxetine, sertraline, and chlorpromazine. However, these trials were unsuccessful and caused numerous side effects. For example, the amphetamine-dextroamphetamine and methylphenidate worsened his DMDD, while the aripiprazole caused akathisia. Escitalopram was noted to cause anxiety. The patient's mother does not recall the duration of therapy for any of these medications or the dosages. He has been admitted to multiple inpatient psychiatric facilities locally and was terminated from these facilities due to persistent aggressive behavior, which concerned staff to the point that they felt they were unable to keep him safe. Furthermore, the patient has been attending virtual therapy sessions due to the COVID-19 pandemic. The patient also had difficulties attending his primary care appointments due to ongoing transportation issues. Currently, the patient is being treated at an out-of-state psychiatric facility.

## Discussion

In the context of our patient, his persistent, medication-resistant aggression, as evident by his trial and failure of several medications, is most secondary to his ADHD and DMDD. Between the ages of 2 and 17 years, DMDD has a prevalence between 0.8% and 3.3% [7]. Approximately 67% of the individuals with DMDD have another comorbid condition, such as ADHD [7]. ADHD has a prevalence of approximately 3.74% in the population, which has increased from 2.96% between 2007 and 2016 [8]. These outcomes are linked to and exacerbated by rising levels of poverty, hospitalization, sexually transmitted diseases, and ACEs [7,8]. As a result, it is imperative to give patients appropriate psychiatric care that is consistent, meets the needs of the patient, and is sustainable over time. Though there are numerous resources available for individuals afflicted with various mental health conditions, mental health resources are not evenly distributed, contributing to a disparity of mental healthcare. Patients living in rural areas often commute several hours to not only access mental health services but also obtain their psychotropic medications [8]. Impoverished individuals must contend with complicating factors, dealing with both the stigma of mental health and living in poverty, resulting in decreased use of mental health services [8]. Additional difficulties include the inability to pay for mental health services and the stress associated with affording basic necessities, e.g., food and shelter. Though the COVID-19 pandemic has made health care more accessible in some respects as evident by the rising usage of telehealth, it has also complicated things as well since many individuals became unemployed during this time, further exacerbating barriers that make accessing mental health services difficult.

This case ultimately highlights the need to address the growing challenges of adverse mental health outcomes, especially among individuals living in underserved areas, where healthcare access is limited. It is imperative to take a preventative approach rather than a reactive approach that aims at immediately increasing amount of mental health providers, something which is difficult to achieve at this time due to the limited number of residency spots available. For physicians, training on behavioral and psychotropic medication should be included in residency curricula, and efforts should be made for primary care providers to have access to continuing medical education that can increase the level of comfort with managing these conditions. This is especially important in underserved rural and urban areas, where pediatric mental healthcare access is limited, such as in our state [8]. Physicians should extend their capabilities and offer flexibility, especially in practice locations such as rural settings, i.e., by offering virtual appointments. This is especially important if the primary care physician is the only provider within the vicinity. Furthermore, providers should become familiarized with the various psychosocial stressors that contribute to adverse mental health outcomes and be able to provide appropriate resources to address these factors. These stressors include limited health care access, ACEs, and other social determinants of health that can be screened for early in life. Given the patient’s medical history of being on several medications, this case highlights the need for genetic testing as a part of the workup for psychiatric conditions that are medication-resistant, as evident by multiple failed trials of medications. It is important to note that genetic testing is not readily available in Nevada nor is it covered by most public health insurance programs such as Medicaid.

## Conclusions

Identifying and treating mental health conditions early in life can prevent an individual from having social difficulties later on in life. In the context of aggression, providing the appropriate interventions is key to preventing an individual from engaging in harmful activities, such as homicides and other criminal activities. It is extremely important that screening for ACEs is performed and addressed early to help alleviate these well-known factors that contribute to the development and persistence of aggressive behaviors. Furthermore, individuals living in areas with limited resources or those suffering from poverty face several challenges that prevent them from accessing quality healthcare services. It is imperative that current primary care physicians, such as pediatricians and family medicine physicians, in underserved areas receive training necessary to recognize and address relevant psychosocial contributors. Ultimately, the overarching solution is to improve access to mental healthcare services, especially for low socioeconomic status populations, who may otherwise suffer the continuing sequelae of untreated psychiatric conditions.
